# Depression, stress, and anxiety versus internet addiction in early and middle adolescent groups: the mediating roles of family and school environments

**DOI:** 10.1186/s40359-024-01659-z

**Published:** 2024-04-03

**Authors:** Maryam Aziz, Khansa Chemnad, Sanaa Al-Harahsheh, Azza O. Abdelmoneium, Ahmed Baghdady, Raian Ali

**Affiliations:** 1 College of Science and Engineering, Hamad Bin Khalifa University, Doha, Qatar; 2 World Innovation Summit for Health, Qatar Foundation, Doha, Qatar; 3 Doha International Family Institute, Qatar Foundation, Doha, Qatar; 4 World Innovation Summit for Education, Qatar Foundation, Doha, Qatar

**Keywords:** Adolescence, Anxiety, Depression, Internet addiction, Stress

## Abstract

**Background:**

Family and school environment play a crucial role across the different developmental stages of adolescence. This paper investigates the potential mediating role of family and school environments in the relationship between the three psychosocial predictors of depression, anxiety, stress, and Internet addiction (IA). Specifically, it focuses on the two stages of early and middle adolescence.

**Methods:**

The study involved a survey of 407 adolescents from Qatar, comprising 250 early adolescents and 157 middle adolescents. Inclusion criteria for the study included adolescents between the ages of 10 to 17 years old, residents of Qatar and studying in a Qatar-based school. To assess the constructs of the three psychosocial predictors, IA, family environment, the study utilized the Depression, Stress, and Anxiety Scale (DASS), the Internet Addiction Diagnostic Questionnaire (IADQ), and the Brief Family Relationship Scale, respectively. School environment was measured using questions from the “Health Behavior in School-aged Children: WHO Collaborative Cross-National survey/study (HBSC) 2013–2014. The study applied standard mediation analysis between the DASS components and IA with family and school environment as the mediators.

**Results:**

Results from the mediation analysis reveal insights into the relationships between psychosocial predictors and IA. The findings indicate that family and school environments partially mediated the relationship with regards to depression, stress, and anxiety in early adolescents. In middle adolescents, family environment partially mediated the relationship with depression and stress and fully mediating the relationship with anxiety. Meanwhile, school environment only exhibited partial mediation in the relationship with anxiety in middle adolescence.

**Conclusions:**

These results highlight the crucial role parents and schools play in addressing problematic technology usage that develops as a response to depression, anxiety, and stress among adolescents. Moreover, the study reveals nuances in the mediating role of family and school environment in early and middle adolescence. This highlights the evolving nature of these influences across the different stages of development. Notably, this study contributes to the literature by moving beyond the conventional focus on the so-called WEIRD population, and offering valuable insights from a region that is underrepresented in current research.

## Background

The excessive use of the Internet in daily lives is leading to growing public concerns towards adolescent Internet Addiction (IA). High prevalence rates of adolescent IA have been estimated in several countries including 26.5% in China [1], 20.6% in the United States [2], 23.7% in Japan [3] and 17.7% in Turkey [4]. The direct comparisons between these rates should be approached with caution due to subtle differences in the time of study, sampling methods, and measurement tools used in each research. While IA is not officially recognized as a mental disorder by the American Psychiatric Association (APA) [5], gaming disorder is formally included in the 11th version of the International Classification of Diseases (ICD-11) by WHO in 2018 [6]. As of yet, no clear and definitive definition exists of IA [7], however, several broad definitions have been mentioned in literature. IA, also known as “problematic Internet use”, “compulsive Internet use”, and “pathological Internet use” is defined as the excessive or uncontrollable use of the Internet leading to symptoms of withdrawal, tolerance, and negative psychological concerns by Young [8]. Lai & Kwan [9] gave an alternative perspective on IA defining it as “the excessive use of the Internet that causes disturbances or harm to the individual”.Click or tap here to enter text. Furthermore, previous literature has also widely debated on the constructs of IA. Baggio et al. [10] found problematic online behaviors manifested through problematic Internet use, occurred as separate but related issues. Despite no official diagnostic criteria existing for IA, Young [8] defined the diagnostic criteria for IA based on the extreme gambling criteria suggested in DSM-IV. The four characteristics of addictive behaviors mentioned in DSM-IV: excessive use, withdrawal, tolerance, and adverse psychological consequences are frequently observed in internet-addicted individuals [11]. As such, the Young’s Internet Addiction Scale is an adaptation of the DSM-IV symptoms for addictive behaviors. Adolescents, particularly, are vulnerable to IA as the sensitivity of their developing brains to signals of excitement causes them difficulties in controlling their Internet use [12]. IA in adolescents has also been linked to other maladaptive behaviors such as aggressiveness [13, 14], impulsivity [14] and suicidal behavior [15]. This makes it crucial to identify and understand the factors of IA and their underlying mechanisms.

### Adolescent IA and psychosocial predictors

IA has been repeatedly associated with negative psychosocial conditions in adolescents. Several studies have demonstrated that depression [16–18], stress [17–19], and anxiety [16, 17] were positively related with IA in adolescents. IA can be both a result of a psychological state or condition and a way to mitigate and cope with it. Adolescents may turn to the Internet as a way to cope with negative emotional problems [20]. This may be explained by the mood enhancement hypothesis that states that individuals facing psychosocial problems are more likely to turn to recreational activities including technology use to alleviate their mood [21]. Online interactions may also provide a sense of comfort with reduced perceived threat compared to face-to-face interactions due to the anonymity involved. While adolescents’ sensitivity to excitement may cause them to feel temporary happiness and relief when using the Internet, they may not understand that it is a means of escape rather than the solution to their emotional state. The temporary change from negative to positive emotions with internet use may lead the adolescents to assume that increased internet use leads to increased happiness. However, they may end up being trapped in the cycle of continued Internet use to lessen negative emotions and improve positive emotions without realizing that they no longer experience the same level of change from negative to positive emotion over time [22].

### WEIRD vs. Non-WEIRD Population: sampling considerations

Most literature in psychology relies on the western, educated, industrialized, rich, and democratic (WEIRD) population creating the so-called replication crisis in psychology and sampling bias [23]. In contrast, studies aimed at the non-WEIRD population are limited. Henrich et al. [23] argues that the WEIRD population constitutes to about 12% of the world’s population hence the results from the WEIRD-dominated population cannot be generalized to the rest of the world. Since adolescent IA is a global concern with Asian countries reportedly having higher prevalence rates compared to Western countries [24], it is essential to conduct studies inclusive of the non-WEIRD population. Studies show that adolescents IA is impacted by academic pressure [25], cultural differences [26], and social aspects [11]. It is important to note that the WEIRD and non-WEIRD population may differ in these educational, cultural, and social domains. This raises the need to study adolescent IA with a focus on the non-WEIRD population. Our study adds to the literature on non-WEIRD population and adolescent IA by utilizing a sample from the population resident to Qatar.

### Developmental changes and adolescence period

During the adolescence period, individuals experience major developmental changes including physical, emotional, and cognitive transitions. This phase also involves the development of one’s ability to reflect on their own and other’s behaviors with strong reflective functioning being a protective factor against risky behaviors [27]. These developmental changes differ amongst the various stages of adolescence. Early adolescence period is marked by dramatic physical, cognitive, and social changes. Along with the shift from childlike features to a more adult appearance, early adolescents also see a change in their social relationships and cognitive thinking [28]. Social relationships in this period expand beyond their families to include peer groups. The early adolescence period also involves individuals experiencing rapid cognitive growth and struggling with their sense of identity and ability to regulate emotions [29]. The middle adolescence period sees a continuing physical growth along with an increasing need to find one’s unique competence, which is the abilities and interests that become a part of their identity [30]. Past literature has suggested that adolescents’ identity development may be linked to the development of problematic behaviors [31].

### Socioecological influences: family and school environment

The socioecological model by Bronfenbrenner [32] states that developing individuals are influenced by the various levels of ecological systems surrounding them and their interactions with these systems. The model places parents and schools as the central part of an individual’s immediate environment, that is one that has the most influence on them during the development phase. According to the attachment theory by Bowlby [33], when parents provide children with a secure environment, they tend to be more confident, and develop better social and emotional skills. With the onset of adolescence period introducing difficult developmental changes, family environment can play a center role towards the development of problem behaviors [34]. Having a negative family environment prone to parental conflicts, hostility, and inconsistency can negatively impact adolescent mental health. A study by Wang et al. [35] found family dysfunction was positively related to depression and anxiety in Chinese early adolescents. Another longitudinal study on Chinese adolescents found that family dysfunction was positively associated with anxiety levels in adolescents [36]. Family environment also plays an essential role in how adolescents develop effective coping strategies [37]. A survey on adolescents in China found the quality of family relationships posed as risk factor for adolescent Internet addiction [1]. Another survey conducted on early adolescents in Central Taiwan found family functionality and depression as predictors of adolescent IA where adolescents with poor family atmosphere and high depression were more prone to Internet addiction [38]. In addition, a study by Aziz et al., [39] surveyed parents in Qatar and looked at the interplay between adolescent IA and the family-related factors of parents’ IA, frequency of parents’ serious arguments with adolescent on excessive Internet use and parenting styles. Their results identified three different interactions between adolescent IA and the family factors namely assertive, aggressive, and lenient interactions. Research on the relation between parental IA and adolescent IA in Chemnad et al. [40] utilized the same dataset used in [39] and showed that adolescents were highly likely to be dependent Internet users when their parents were also dependent Internet users showing the important role of family in adolescent IA. This leads us to propose the following hypothesis:

#### H1

Family environment mediates the relationship between depression, stress, and anxiety (DAS), and IA in adolescents.

Adolescents spend a significant amount of time in schools, making it a key environment setting that can strongly influence their psychological and emotional well-being [41]. Factors of school environment, such as academic pressure, have been shown to impact adolescent mental health negatively. For instance, a study by Frӧjd et al. [42] revealed that middle adolescents experiencing problems with school performance and assignments reported higher levels of depression. Similarly, a survey conducted by Ogilvie et al. [43] found significant associations between school-related issues, such as school difficulties, engagement, and avoidance, and psychological and emotional problems in adolescents. Academic challenges, including struggling with homework completion and unnecessary pressure to keep up with the class, can lead to frustration, feelings of inadequacy, and contribute to elevated levels of stress and anxiety.

School environment has also been associated with maladaptive behavior in adolescents. The study by Chemnad et al. [44] indicated that a positive school environment was linked to lower levels of IA among adolescents, suggesting that a supportive and nurturing school setting can be beneficial for their well-being. On the other hand, a cross-sectional study by Zhai et al. [45] found that adolescents who perceived their school climate negatively were more likely to report higher levels of Internet addiction. This highlights the significant role schools play in shaping adolescent behavior and the importance of creating a positive and conducive learning environment to promote healthier outcomes. Thus, we propose the following hypothesis:

#### H2

School environment mediates the relationship between depression, stress, and anxiety (DAS), and IA in adolescents.

### Research gap and study rationale

On the context of adolescent IA, several studies reported levels of IA differed amongst the different age groups of adolescents with IA level increasing as age progresses in adolescents [19]. While both early and middle adolescents are vulnerable to IA, their different developmental stages may lead them to being exposed to different IA related risk and protective factors. For example, a study by Badenes-Ribera et al. [46] found levels of Facebook addiction in early adolescents was influenced by parental attachment whereas in middle adolescents Facebook addiction levels were impacted by peer relationships. The middle adolescence period also sees an increasing reliance on peer groups and a need to separate from familial relations [29]. Technology access also differs among early and middle adolescents. A survey conducted on late adolescents in United States found almost 16% reported receiving smartphones in early adolescent stage whereas approximately 61% reported receiving smartphones in middle adolescence period [47]. This makes it crucial to study early and middle adolescents individually and the impact that different risk factors of IA may have on them. By studying them independently, interventions and prevention approaches can be effectively tailored to each adolescent phase taking into consideration the differences due to their developmental changes, relationship dynamics and family influences.

Family and school environment play a central role in an adolescent’s life and are key to understanding adolescent IA and mental health. A major gap in the literature relates to the limited studies on the role of family and school environment when discussing DAS and adolescent IA. Moreover, prior studies did not make a clear distinction between the early and middle adolescence stages in that context. In addition, most literature on adolescent IA predominantly utilized a WEIRD sample whereas limited research is conducted on the non-WEIRD sample. Hence, our research aims to add to the literature on DAS and adolescent IA by studying the mediating role of family and school environment in a sample of early and middle adolescents that are resident to Qatar.

## Methods

### Participants

Participants for this study were recruited through an online survey administered using SurveyMonkey. The link to the survey was shared with the students at 16 schools selected randomly. The survey period lasted 3 months between March 2022 and May 2022. Prior approval was obtained from the Institutional Review Board (IRB) of Hamad Bin Khalifa University (No. QBRI-IRB 2021-05-094) and the study was carried out according to the guidelines stated in the Declaration of Helsinki. In addition, school permissions were also acquired to distribute the survey and informed consent was obtained from both parents and adolescents. Participation in the study was voluntary.

Inclusion criteria for this study comprised of adolescents between the ages of 10 to 17 years old, residents of Qatar and studying in a Qatar-based school. Participants that did not meet the inclusion criteria were excluded from the study. We did not include the technology penetration criteria in our inclusion criteria as technology penetration in Qatar is already high bordering at 99% [48]. Of the 586 participants who responded, those who gave incomplete and hasty answers were removed. As such, 407 participants were included in the final study which met the criteria of 5% margin of error at the 95% confidence interval.

### Measures

The survey collected information regarding participants’ demographics, digital technology usage and IA. Data regarding participants’ physical and psychological health status were also gathered. The survey was administered in both English and Arabic. The Arabic survey was generated using the back-translation method [49] to ensure the quality of translation and the preservation of the original meaning is maintained.

#### Internet Addiction Diagnostic Questionnaire (IADQ)

The Internet Addiction Diagnostic Questionnaire (IADQ) was used to assess adolescent IA level. The IADQ comprised of eight closed-ended questions (yes/no) where the IA score is the total number of questions the participant responded to in affirmative. Each question represents a different symptom of IA that are preoccupation with internet, tolerance, unsuccessful efforts to control Internet use repeatedly, withdrawal, staying online longer than intended, risk/loss of relationships and opportunities because of the Internet use, lies to conceal the extent of involvement and dysfunctional coping. Participants were asked to answer the questionnaire based on their non-essential internet use, (non-business or non-academic use). The IADQ total score ranges between 0 and 8, where Young [8] suggests that a score of 5 or higher classifies the participant as a dependent internet user. In line with the suggestion by Young [8], we classified participants who scored 5 or above as dependent users and below 5 as non-dependent users. Previous literature on IADQ states the Cronbach’s alpha to be within the range of 0.60 and 0.72 [50]. The Cronbach’s alpha for the present sample was 0.64.

#### Depression, anxiety, stress scale – 21 (DASS-21)

The Depression, Anxiety, Stress Scale – 21 items is a set of three self-report scales that are used to assess the degree of depression, anxiety, and stress in individuals [51]. The three subscales each have seven items that measure a negative emotional symptom. The depression scale rates symptoms of dysphoria, helplessness, life devaluation, self-deprecation, loss of interest, anhedonia, and lethargy. The autonomic arousal, skeletal muscle effects, situational anxiety, and subjective sensation of anxious affect are all measured by the anxiety scale. The stress scale evaluates issues with relaxation, nervousness, irritability, overreacting, and impatience. The scores for the relevant questions are added up to determine the scores for depression, anxiety, and stress and then scaled up by 2. Each subscale ranges from 0 to 42 and the cutoff scores for the severity categories (normal, mild, moderate, severe, and extremely severe) are unique to each subscale. Cronbach’s alpha for depression scale was found to be 0.94 in literature, 0.87 for the anxiety scale and 0.91 for the stress scale [52]. For the present study, the Cronbach’s alpha for depression, anxiety and stress scale was determined to be at 0.88, 0.87, and 0.85 respectively which indicates a very good reliability [53].

#### Brief family relationship scale (BFRS)

The Brief Family Relationship Scale is a 16-item scale that measures three dimensions of family functioning that is cohesion, expressiveness, and conflict. The BFRS is used to examine youth perceptions of family. The scale uses a 3–point Likert scale where 1 = not at all, 2 = sometimes, and 3 = a lot. The total score is the sum of the 16 questions and participants can accumulate a maximum possible score of 48 where the higher the score the less family functioning is experience. The Cronbach alpha was reported as 0.88 [54] in literature whereas for the present study it was found to be 0.89, a very good level of reliability [53].

#### School environment

The school environment variable was measured using questions from the “Health Behavior in School-aged Children: WHO Collaborative Cross- National survey/study (HBSC) 2013–2014” [55]. The HBSC is an international collaboration project between research groups across North America and Europe aimed at understanding adolescents’ health and wellbeing behaviors. The questions were adapted from the section on school settings in HSBC. Three items assessed the school environment: (1) “how do you feel about school at present?”, (2) “how pressured do you feel by the schoolwork/homework you have to do?”, and (3) “how much of a problem have you had getting your schoolwork/homework done on time?”. Responses for the school feeling were 1= “I don’t like it at all”, 2= “I don’t like it very much”, 3= “I like it a bit” and 4= “I like it a lot”. The possible responses for school pressure were 1= “A lot”, 2= “Some”, 3= “A little” and 4= “Not at all”. For schoolwork problems, the possible responses were 1= “A serious problem”, 2= “A considerable problem”, 3= “Some problem” and 4= “No problem”.

### Statistical analysis

Descriptive statistics were computed for all variables. The statistical analysis for this study was performed using JASP 0.16.3 [56]. Exploratory factor analysis was employed with an orthogonal varimax rotation to discover the underlying structure of the school environment variables. The number of factors to retain was determined by the following criteria: (1) The scree plot needed to align with the number of factors extracted, and (2) the resulting factors lead to a meaningful interpretation. Mediation analysis was conducted using the standard mediation method available in the JASP software. JASP utilizes the product of coefficient approach [57] to implement mediation analysis. This method provides more robust statical power and accurate Type I error rates. The standardized values for DAS, family environment, school environment and internet addiction scores were used for the mediation analysis. It is also worth noting that typical mediation analysis means that changes in the predictor, DASS components, lead to changes in the mediator, family, or school environment, which in turns leads to changes in the outcome, IA. However, in our specific research context, we are employing an approach known as exploratory mediation analysis [58] where the mediator explains the relation between the predictor and the outcome without implying causality and does not vary with changes in the predictor. The mediator in this analysis is used to better understand and describe the relations between the predictor and outcome without establishing any causality. For instance, in the case of school environment, adolescents may resort to using the Internet excessively if the environment is not supportive. On the other hand, a supportive environment may help them utilize better coping strategies. Statistical significance for this study was accepted at *p* < .05.

## Results

The final sample had 407 participants with the mean age of 13.2 years (SD = 1.24). The sample consisted of 330 (81.1%) females and 77 (18.9%) males. Two of the schools that highly responded to the survey were predominantly female schools. The sample also included 250 early adolescents (10–13 years old) and 157 middle adolescents (14–17 years old). Of the middle adolescents, only four participants were aged 17 years.

### Descriptive statistics

Table 1 summarizes the descriptive statistics of the sample for this study. The participants were all residents of Qatar. The sample included 75.2% Arabs, 16.5% from Eastern backgrounds, and 8.3% from Western backgrounds. Additionally, the sample comprised 70% non-Qataris and 30% Qataris.

**Table 1:** Descriptive statistics of sample variables

Parameters	Early		Middle	
	Mean	Std. dev.	Mean	Std. dev.
School feeling	3.08	0.77	2.92	0.88
School pressure	2.36	0.92	2.10	0.91
Schoolwork problem	2.94	0.87	2.92	0.87
BFRS	38.65	6.60	39.47	5.83
IADQ	3.26	1.90	3.54	2.16
Depression	13.13	11.36	13.50	11.69
Stress	13.94	10.66	14.29	10.40
Anxiety	14.14	11.65	13.59	11.70
**Internet Addiction prevalence**	**Early**		**Middle**	
	**n**	**%**	**n**	**%**
Addicted (5 + Yes’s)	64	25.60	57	36.31
Non-addicted	186	74.40	100	63.69

### Factor analysis of school environment

Exploratory factor analysis on the sample resulted in the grouping of the three items into a single variable of school environment. We measured the Kaiser-Meyer-Olkin (KMO) to measure the sample adequacy for factor analysis. The overall KMO measure was 0.66 with individual KMO measures between the range of 0.64 and 0.71. As per [59], the KMO measures fall into the categories of ‘mediocre’ to ‘middling’. Bartlett’s test of sphericity was statistically significant (*p* < .001), indicating that the variables are unrelated and suitable for factor analysis.

The factor analysis resulted in one component which accounted for 44% of the variance. The resulting scree plot also aligned with the one-factor solution. This one factor was interpreted as representing the school environment. Table 2 presents the component loadings and uniqueness of the factor. In addition, the derived school environment variable was calculated by taking the average of the individual items.

**Table 2:** Factor analysis of school environment

		Factor 1		Uniqueness	
School pressure		0.71		0.50	
Schoolwork problem		0.70		0.52	
School feeling		0.58		0.67	

### Correlation between sample variables for early and middle adolescents

As shown in Tables 3 and 4, all variables are correlated in the analysis for early and middle adolescents respectively.

**Table 3:** Correlation between the sample variables for early adolescents

Variable	Pearson’s r					
	1	2	3	4	5	6
1. IADQ	—					
2. FRQS	-0.39***	—				
3. School environment	-0.35***	0.45***	—			
4. Stress (DASS)	0.52***	-0.49***	-0.49***	—		
5. Depression (DASS)	0.50***	-0.48***	-0.49***	0.83***	—	
6. Anxiety (DASS)	0.49***	-0.38***	-0.42***	0.80***	0.73***	—

* *p* < .05, ** *p* < .01, *** *p* < .001

**Table 4:** Correlation between the sample variables of middle adolescents

Variable	Pearson’s r					
	1	2	3	4	5	6
1. IADQ	—					
2. FRQS	-0.48***	—				
3. School environment	-0.32***	0.47***	—			
4. Stress (DASS)	0.45***	-0.51***	-0.45***	—		
5. Depression (DASS)	0.44***	-0.56***	-0.49***	0.82***	—	
6. Anxiety (DASS)	0.32***	-0.42***	-0.45***	0.77***	0.72***	—

* *p* < .05, ** *p* < .01, *** *p* < .001

### Mediation analysis of family environment in early and middle adolescents

The mediation analysis included stress, depression and anxiety as the independent variables, and IA as the dependent variable. The family environment variable was included as a potential mediator. The model met the assumptions of continuous variables, correlation between dependent, mediating, and independent variables, multicollinearity, and outliers [60].

**Fig. 1:**
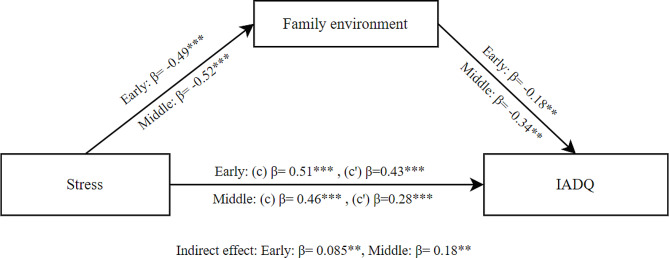
Mediation model between stress and IADQ through family environment for early and middle adolescents; (c) Total effect, (c’) Direct effect; * *p* < .05; ** *p* < .01; ***p < .001

The mediation model of stress as independent variable, family environment as mediator and IA as dependent variable showed a significant total effect of stress on IA (β = 0.51, SE = 0.054, *p* < .001). The direct effect was also significant (β = 0.43, SE = 0.061, *p* < .001). Additionally, the indirect effect of family environment was significant (β = 0.085, SE = 0.031, *p* = .007). These findings indicate that family environment partially mediated the effect of stress on IA for early adolescents (see Fig. 1).

For middle adolescents, the mediation model showed a significant total effect of stress on IA (β = 0.46, SE = 0.073, *p* < .001). The direct effect was also significant (β = 0.28, SE = 0.080, *p* < .001). Additionally, the indirect effect of family environment was significant (β = 0.18, SE = 0.047, *p* < .001). These findings indicate that family environment partially mediated the effect of stress on IA for middle adolescents as shown in Fig. 1.

**Fig. 2:**
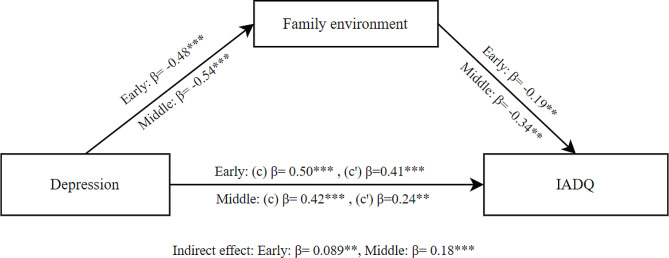
Mediation model between depression and IADQ through family environment for early and middle adolescents; (c) Total effect, (c’) Direct effect; * *p* < .05; ** *p* < .01; ***p < .001

The mediation results for depression as independent variable, family environment as mediator and IA as dependent variable showed a significant total effect of depression on IA (β = 0.50, SE = 0.054, *p* < .001) in early adolescents. The direct effect was also significant (β = 0.41, SE = 0.061, *p* < .001). Additionally, the indirect effect of family environment was significant (β = 0.089, SE = 0.031, *p* = .004). These findings indicate that family environment partially mediated the effect of depression on IA for early adolescents as shown in Fig. 2.

For middle adolescents, the mediation results showed a significant total effect of depression on IA (β = 0.42, SE = 0.069, *p* < .001). The direct effect was also significant (β = 0.24, SE = 0.079, *p* = .003). Additionally, the indirect effect of family environment was significant (β = 0.18, SE = 0.049, *p* < .001). These findings indicate that family environment partially mediated the effect of depression on IA for middle adolescents (see Fig. 2).

**Fig. 3:**
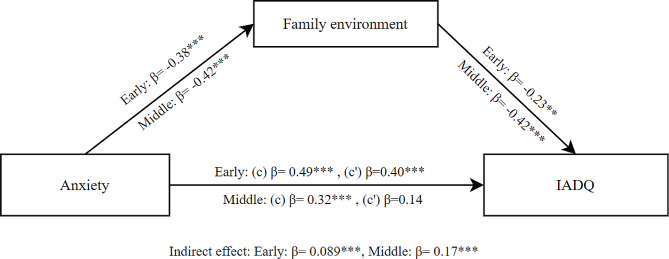
Mediation model between anxiety and IADQ through family environment for early and middle adolescents; (c) Total effect, (c’) Direct effect; * *p* < .05; ** *p* < .01; ***p < .001

The mediation results for anxiety as independent variable, family environment as mediator and IA as dependent variable showed a significant total effect of anxiety on IA (β = 0.49, SE = 0.055, *p* < .001) in early adolescents. The direct effect was also significant (β = 0.40, SE = 0.058, *p* < .001). Additionally, the indirect effect of family environment was significant (β = 0.089, SE = 0.026, *p* < .001). These findings indicate that family environment partially mediated the effect of anxiety on IA for early adolescents as shown in Fig. 3.

For middle adolescents, the mediation results also showed a significant total effect of anxiety on IA (β = 0.32, SE = 0.075, *p* < .001). However, the direct effect was not significant (β = 0.14, SE = 0.076, *p* = .063). The indirect effect of family environment was significant (β = 0.17, SE = 0.044, *p* < .001). These findings indicate that family environment fully mediated the effect of anxiety on IA for middle adolescents as shown in Fig. 3.

### Mediation analysis of school environment in early and middle adolescents

The mediation analysis included stress, depression and anxiety as the independent variables, and IA as the dependent variable. The school environment variable was included as a potential mediator. The model met the assumptions of continuous variables, correlation between dependent, mediating, and independent variables, multicollinearity, and outliers.

**Fig. 4:**
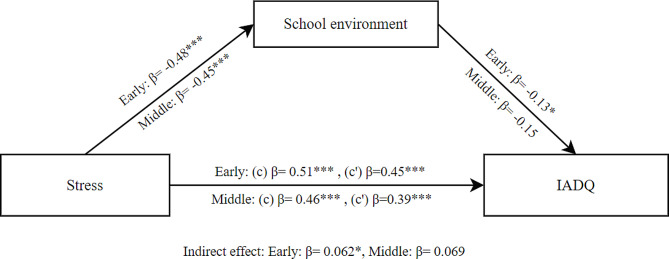
Mediation model between stress and IADQ through school environment for early and middle adolescents; (c) Total effect, (c’) Direct effect; * *p* < .05; ** *p* < .01; ***p < .001

The mediation model of stress as independent variable, school environment as mediator and IA as dependent variable showed a significant total effect of stress on IA (β = 0.51, SE = 0.054, *p* < .001) in early adolescents. The direct effect was also significant (β = 0.45, SE = 0.061, *p* < .001). We also found the indirect effect of school environment was significant (β = 0.062, SE = 0.030, *p* = .040). These findings indicate that school environment partially mediated the effect of stress on IA for early adolescents as shown in Fig. 4.

For middle adolescents, the mediation model showed a significant total effect of stress on IA (β = 0.46, SE = 0.073, *p* < .001). The direct effect was also significant (β = 0.39, SE = 0.080, *p* < .001). However, the indirect effect of school environment was not significant (β = 0.069, SE = 0.037, *p* = .065). These findings indicate that school environment did not mediate the effect of stress on IA for middle adolescents.

**Fig. 5:**
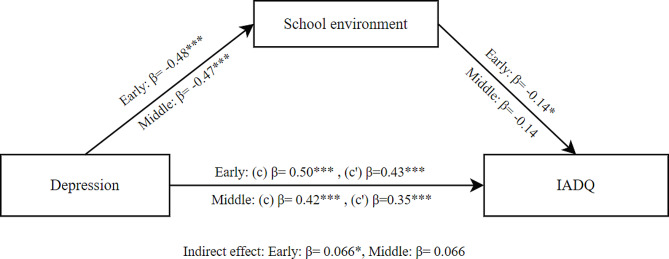
Mediation model between depression and IADQ through school environment for early and middle adolescents; (c) Total effect, (c’) Direct effect; * *p* < .05; ** *p* < .01; ***p < .001

The mediation results for depression as independent variable, school environment as mediator and IA as dependent variable showed a significant total effect of depression on IA (β = 0.50, SE = 0.054, *p* < .001) in early adolescents. The direct effect was also significant (β = 0.43, SE = 0.061, *p* < .001). Additionally, the indirect effect of school environment was significant (β = 0.066, SE = 0.031, *p* =.033). These findings indicate that school environment partially mediated the effect of depression on IA for early adolescents as shown in Fig. 5.

The mediation results for middle adolescents showed a significant total effect of depression on IA (β = 0.42, SE = 0.069, *p* < .001). The direct effect was also significant (β = 0.35, SE = 0.079, *p* < .001). However, the indirect effect of the school environment was not significant (β = 0.066, SE = 0.040, *p* =.094). These findings indicate that school environment did not mediate the effect of depression on IA for middle adolescents.

**Fig. 6:**
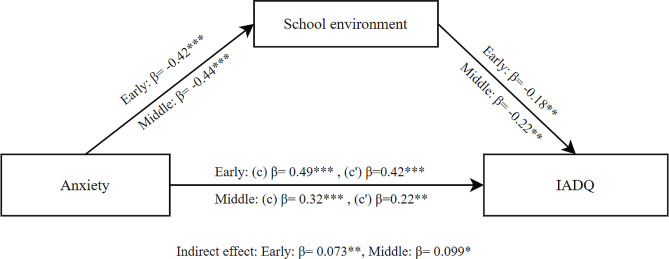
Mediation model between anxiety and IADQ through school environment for early and middle adolescents; (c) Total effect, (c’) Direct effect; * *p* < .05; ** *p* < .01; ***p < .001

The mediation results for anxiety as independent variable, school environment as mediator and IA as dependent variable showed a significant total effect of anxiety on IA (β = 0.49, SE = 0.055, *p* < .001) in early adolescents. The direct effect was also significant (β = 0.42, SE = 0.060, *p* < .001). Additionally, the indirect effect of school environment was significant (β = 0.073, SE = 0.027, *p* =.007). These findings indicate that school environment partially mediated the effect of anxiety on IA for early adolescents as shown in Fig. 6.

The mediation results for middle adolescents showed a significant total effect of anxiety on IA (β = 0.32, SE = 0.075, *p* < .001). The direct effect was also significant (β = 0.22, SE = 0.082, *p* =.009). Additionally, the indirect effect of school environment was significant (β = 0.099, SE = 0.040, *p* =.013). These findings indicate that school environment partially mediated the effect of anxiety on IA for middle adolescents as shown in Fig. 6.

## Discussion

This study explored the underlying role of family and school environment in the relationship between depression, stress, and anxiety and IA in early and middle adolescents. The main findings of this study are threefold. First, family environment mediates the relationship between DAS and IA in early and middle adolescents. Second, school environment partially mediates the relationship between DAS and IA in early adolescents. Third, school environment partially mediates the relationship between anxiety and IA in middle adolescents but does not mediate the relationship between depression and IA and between stress and IA.

In line with our first hypothesis, we observed an indirect effect of DAS on adolescent IA through the family environment in both early and middle adolescents. This implies the significant role of family environment in prevention and intervention efforts for adolescent IA. Further, our findings revealed that family environment fully mediates the relationship between anxiety and IA in middle adolescents, indicating that anxiety does not directly impact IA but rather affects it indirectly through the family environment. These results suggest that family dynamics can influence adolescents’ susceptibility to DAS and subsequent engagement in IA.

Our findings align with the Bronfenbrenner model [32] suggesting that being in a negative family environment can lead to adolescents looking for alternative sources of emotional support and developing maladaptive coping mechanisms such as IA. Adolescents living in a healthy environment rely on their parents for emotional support, reducing the need for maladaptive coping strategies. The attachment theory proposed by Bowlby [33] also supports this idea, as it suggests that healthy parental attachments foster the development of healthy coping skills in adolescents. Wang et al. [61] reported that good parent-adolescent relationship was positively related with adolescents’ emotion regulation ability, which in turn was negatively related with IA. Conversely, living in a dysfunctional family environment may lead to an impaired sense of security, increasing the risk of psychological and behavioral problems [20]. Factors like parental conflict, family discord, and lack of communication may magnify the impact of DAS on adolescents, potentially driving them towards excessive internet use as a means of escape, and ultimately contributing to the development of IA. This behavior is commonly seen in other addictions as well for example, a study conducted in Qatar found a bidirectional relationship between family relationships and adolescent substance addiction that is adolescents living in dysfunctional family environment may resort to substance addiction which may then further negatively impact the family environment [62].

Our findings partly support our second hypothesis indicating that the school environment partially mediated the relationship between DAS and IA in early adolescents. However, for middle adolescents, the school environment only partially mediated the relationship between anxiety, without showing significant effects on depression and stress with IA. These findings emphasize the nuanced role of the school environment during different developmental stages in adolescence.

During early adolescence, students undergo significant academic transitions, moving from primary to middle school [63]. This transition along with the increased hormonal changes and social pressures may impact the mental health struggles of early adolescents. They may be more susceptible to external stressors such as academic pressure and schoolwork difficulties. Furthermore, early adolescents disengaged from school and experiencing negative school feelings, such as a lack of belonging and support, are at a higher risk for psychological distress and involvement in risky behaviors [64]. A survey carried out by Oberle [65] revealed a direct relation between a supportive classroom environment and the emotional well-being of early adolescents. The connection was also influenced indirectly through positive social interactions and self-perception. Early adolescents also lack effective coping skills as they are less trained in evaluating circumstances and formulating effective approaches to manage [66]. This lack of coping skills may make them susceptible to maladaptive behavioral patterns. These findings also align with the Bronfenbrenner model [32] for school environment suggesting that having a school environment that lacks effective support may further reinforce adolescents’ feelings of inadequacy and failure, thus driving them towards seeking relief through the internet and adopt dysfunctional coping mechanisms, one of the symptoms of the IADQ scale. As such, the school environment may either alleviate or aggravate their psychological distress.

On the other hand, middle adolescents show a different pattern. While the school environment still plays a role in mediating anxiety and IA, they have better coping skills and a stronger sense of emotional competence [66]. Having already adjusted to their school environment, they may experience less negative school-related feelings compared to early adolescents. Conversely, middle adolescents encounter a more demanding and competitive learning environment compared to early adolescents. Middle adolescents are confronted with elevated parental expectations and heightened academic stress, factors that can significantly influence their level of academic involvement [67]. Simultaneously, these middle adolescents may also grapple with anxiety stemming from the increased pressure to excel academically as they move from high school to college.

Our results also showed that family and school environment were both negatively related with DASS and IA for both early and middle adolescents. This suggests that a positive and supportive family and school environment is related to lower levels of IA and DAS in adolescents. These results align with previous literature on family and school environment with IA where a negative family and school environment were reported as possible risk factors for adolescent IA [1]. A study on depression and anxiety also found family dysfunction positively predicted depression and anxiety in adolescents [35]. Further, a systematic literature review reported that school environment is related to adolescent mental health [68]. Our results further reinforce the existing body of literature highlighting the impact of family and school environments.

With Qatar having one of the highest internet penetration rates in the world at 99% [48], the culture of convenience in addition to the hot weather during most of the year, leads to people spending most of their indoors. Sedentary behavior in adolescents is also associated with negative psychosocial factors of depression, stress, and anxiety [69]. A study by Al-Kaabi et al. [70] found that almost 34% of Qatari adolescents in secondary schools reported experiencing symptoms of depression. Their results also showed that relationships with family, peers and teachers were significantly related to symptoms of depression. Furthermore, while the majority of Qatar’s population is immigrants, the family-wise cultural differences in family structure and norms between Qataris and other Arab countries is relatively low, and our sample comprised of approximately 75% Arab population (including Qataris). The Arab cultures holds hierarchical family atmosphere where parents and elderly in general are respected and seen as a source of authority and point of reference [71]. These cultural differences may impact the coping strategies adolescents adopt and the level of emotional support available to them in the familial context. Further research is needed to understand the impact the hierarchical family structure may have on mitigating the impact of DAS on IA in adolescents. We also note that the parent’s marital life and support of domestic helps may impact the results however due to sensitivity issues these questions were avoided. Regardless, we have measured the school environment which is based on the UNICEF scale that accounts for the global conditions of family environment and school environment and family environment through the BFRS scale which was validated for Arab population.

Our study is subject to certain limitations that need to be considered when interpreting the findings. Although mediation analysis typically establishes causal relations and is based on a longitudinal research design, our research design adopts a cross-sectional approach, making the establishment of causal relations impossible. Furthermore, we are employing exploratory mediation analysis where the mediator explains the relation between the predictor and the outcome without implying causality and does not vary with changes in the predictor. Additionally, the reliance on self-reported data introduces potential biases that could impact the accuracy of the outcomes. For instance, there is a possibility of recall bias influencing participants’ recollection and reporting of their online activities. A relevant study conducted by Zurbriggen et al. [72] revealed that early adolescents tend to overestimate positive affect, whereas middle adolescents tend to overestimate negative affect when recalling emotional experiences. Another significant source of potential bias is social desirability, wherein respondents might feel inclined to provide socially acceptable responses rather than completely accurate ones. This phenomenon might have affected the accuracy of participants’ reporting, particularly in relation to aspects such as internet addiction and psychological distress. Moreover, it is important to note that our data collection took place during exam periods, which led to a limited number of schools willing to participate. In addition, we received majority of responses from two girls’ schools resulting in approximately 80% female participants. This limited our ability to draw conclusions about potential gender-based differences in adolescents’ internet addiction. While there is limited research on how family and school environment explain the relationship between DAS and adolescent IA, our results should be interpreted with caution when it comes to gender related differences and be seen closer to females than males given the approximately 80% female population. Our sample is also not nationally representative due to certain logistical limitation of administering the survey again in other times of the year as it could lead to bias due to the time of year, and academic calendars amongst other factors.

Despite these limitations, the results of our study have both theoretical and practical implications. From a theoretical perspective, the present study provides empirical evidence of the mediating effect of both family and school environment in the context of internet addiction. Furthermore, our study indicates the distinct impacts that both environments have on psychological distress and internet addiction among early and middle adolescents, particularly regarding anxiety. These findings contribute to the existing literature on adolescent internet addiction and emphasize the necessity of tailoring research to each phase of adolescence. From a practical perspective, our findings demonstrate that both family and school environments are crucial when dealing with adolescent Internet addiction. This corresponds with Bronfenbrenner’s socioecological model, which proposes that individuals are shaped by the immediate environments they inhabit and by the interactions occurring within and between these environments [32]. Abdelrahman et al. [73] reported that parents’ activities with their children and coping strategies were positively related to their children’s mental health in a sample from Qatar that is how parents handle their mental health impacts their children’s mental health as well. Evidence-based prevention and interventions strategies should be rooted in evidence and consider the unique influence of family and school settings, as well as the specific phase of adolescence. A study on the interaction between adolescent IA and family environment factors showed the need to train parents on effective prevention and intervention strategies for adolescent IA [39]. While the family environment bears importance for early and middle adolescents, our findings showed that school environment played a larger role in early adolescents compared to middle adolescents. A systematic literature review on prevention of IA revealed that effective prevention interventions should engage individuals who are integral to the formative environment of at-risk adolescents [74]. This highlights the collective responsibility of parents and schools in cultivating a healthy climate that aids adolescents in developing effective coping mechanisms, thereby mitigating the risk of internet addiction.

Our study did not find a mediating effect of school environment on the relationship between depression and IA or between stress and IA in middle adolescents. The family environment, however, did partially mediate the relationship between depression and stress and adolescent IA. This suggests that the influence of depression and stress on adolescent IA might operate through distinct underlying mechanisms. Future research could explore other potential mediator factors, such as environmental-level factors. For instance, a study by Chung et al. [13] indicated that factors like higher accessibility to PC cafes and Internet game advertising had a greater influence on adolescent internet addiction than family and school environment. Another study conducted in Qatar found time and mood management also impacted internet addiction in their sample [75]. It is also worth noting that this study was from the perspective of adolescents, future research may also include the parental and teacher perspectives to provide a comprehensive view of the relationship between DAS and IA. Our previous work in [39] and [40] shed light into the perspective of parents. We aim to be collecting data from the same household both from the adolescents and their parents to be able to compare perspectives and study how family environment and parent-child relation affect both views. Future research could also employ structural equation modeling to test and establish causal relationships among DAS, family environment, school environment, and adolescent IA. By contrasting different models and incorporating longitudinal and cross-cultural analyses, the study may provide a further nuanced understanding of the complex relationships involved. This would also help in guiding the development of targeted interventions for adolescent internet addiction and mental health. Literature on gender-based differences have no fixed consensus on gender differences in IA. Studies in literature have reported conflicting results with some reporting males as having higher IA levels than females [2, 76] while others reporting females as having higher IA levels [77, 78]. Some have even reported no differences amongst the two genders when it comes to IA [79, 80]. Given that our sample was predominantly female, future studies could benefit from a larger and more balanced sample to investigate the role of gender in adolescent internet addiction. It is also worth noting that adolescents in Qatar can attend private or public schools. Public schools are funded and managed by the government in Qatar whereas private schools are funded by tuition fees and managed by private organizations. Public schools typically follow the Qatari curriculum. Private schools, on the other hand, offer various international curricula that are reviewed by the government. Public schools are generally attended by Qatari citizens. Expatriates are more likely to be enrolled in private schools than public. This is due to the language of instruction in public schools predominantly being Arabic and the access to free education for Qataris. Furthermore, socioeconomic status greatly impacts the literacy across the different school types [81] with higher fees of enrollment to private schools related to more selective curricula and education. While our current research had a sample predominantly from public schools, our future research could look into the impact of the school environment in the different school types on DAS and adolescents’ IA.

## Conclusions

In conclusion, our study provides significant insights into the mediating role of family and school environments on the relationship between DAS and adolescent IA. The findings also highlight the influence of these environments across the two developmental stages of early and middle adolescence.

The family environment emerged as a significant mediator, fully mediating the relationship between DAS and IA. This underscores the importance of family dynamics in shaping adolescents’ susceptibility to psychological distress and subsequent engagement in maladaptive coping mechanisms, such as excessive internet use. Aligning with ecological models and attachment theory, our results suggest that a healthy family environment provides a crucial buffer against IA by fostering positive coping skills and emotional support.

The school environment, while playing a nuanced role, was found to partially mediate the relationship between DAS and IA, with variations between early and middle adolescents. Early adolescents, undergoing significant academic transitions, were more susceptible to negative school-related feelings, contributing to psychological distress and IA. In contrast, middle adolescents, equipped with better coping skills, faced heightened academic stress, revealing a more demanding learning environment.

### Limitations

Despite the valuable insights gained, our study is not without limitations. The cross-sectional design restricts our ability to establish causal relationships, emphasizing the need for future longitudinal studies. The reliance on self-reported data introduces potential biases, including recall bias and social desirability. The predominantly female sample raises questions about gender-related differences, necessitating more balanced participant demographics in future research.

Additionally, our study took place during exam periods, limiting the number of participating schools and potentially influencing responses. The exclusion of certain sensitive questions, such as those related to parental martial life, may have left gaps in our understanding of the family environment’s full impact. Further research is needed to explore the cultural nuances of hierarchical family structures and their potential mitigating effects on the relationship between DAS and IA in adolescents.

### Future research prospects

Looking ahead, future research could delve into additional mediator factors, such as environmental-level variables, to enhance our understanding of the complex dynamics at play. Employing structural equation modeling could help establish causal relationships among DAS, family environment, school environment, and adolescent IA. Exploring cross-cultural and longitudinal analyses would provide a more nuanced understanding of these relationships.

Furthermore, given the observed impact of school environments on IA, future studies could investigate the influence of different school types on psychological distress and IA among adolescents. A more balanced sample that includes both public and private school attendees would contribute to a more comprehensive understanding of the role of the school environment.

### Implications

The study has both theoretical and practical implications. Theoretically, the study contributes to the existing literature by demonstrating the mediating effects of both family and school environments on the relationship between DAS and adolescent IA. The findings also emphasize the importance of tailoring interventions to each adolescence phase. Practically, the study highlights the collective responsibility of parents and schools in addressing adolescent IA. Evidence-based prevention and intervention strategies should consider the influence of family and school settings. Engaging parents in effective prevention strategies and fostering a supportive school environment are crucial components of mitigating internet addiction risks.

## Data Availability

The datasets generated and/or analyzed during the current study are not publicly available but are available from the corresponding author on reasonable request.

## Abbreviations

IA: Internet Addiction
IADQ: Internet Addiction Diagnostic Questionnaire
WEIRD: Western, Educated, Industrialized, Rich, Democratic
DASS: Depression, Anxiety, and Stress Scale
BFRS: Brief Family Relationship Scale
KMO: Kaiser-Meyer-Olkin
SE: Standard Error
